# Bacteriological Pitfalls

**Published:** 1921-07-09

**Authors:** 


### BACTERIOLOGICAL PITFALLS.

The impressive appearance of bacteriological apparatus, together with the past triumphs of this method of investigation, have been responsible for gaining it an undeservedly great reputation for accuracy and reliability, or, more correctly (as de Quincey pointed out), "rely-upon-ability." In our profession there prevails a rather juvenile conception of what science is; we are apt to presume that microscopes and test tubes are indispensable. And, as in other departments of human activity, there is a tendency to pass lightly over facts which go to upset this view. Certainly such facts exist. A professed bacteriologist is not likely to cry stinking fish, and yet Professor Cobbett has discredited the well-known experiments on alimentary pulmonary infection made by Calmette, and confirmed by Sir William Whitla and Professor Symmers at Belfast, on such elementary grounds as the failure to use control subjects; whereby these workers fell into the beginner's blunder of being unaware that town-bred guinea-pigs showed pigmented lungs, whether or not they had been made to swallow Indian ink! What can be said for the scientific exactitude of one who will not compare with the normal when making experiments? Moreover, such comparison will need to be made in a much more exact way than, in pathological laboratories at any rate, has been customary. Of late it has begun to be perceived, what field naturalists have for some time insisted, that even in wild species plain individual differences occur, even in such a stereotyped characteristic as daily habit or life. Now domestication has an acknowledged effect in increasing variability, so that one laboratory rabbit or cavy may differ from another in a way to invalidate many of the rather pontifical pronouncements upon experimental results. The possibilities of this kind of fallacy are well envisaged in a recent article by a Swedish worker.* He shows that the current method of making the dose of infective material proportional merely to the body weight of the animal inoculated is quite misleading. Taking rabbits of three different breeds (a local variety, a blue kind, and Belgian hares), he showed that experiments designed to produce infection turned out quite differently according to breed, although the dose was strictly conformed to body weight. More than this, the age of the animal selected (apparently a point often neglected by bacteriological research workers!) affected matters more seriously than the question of kind, whilst sex could not be pronounced to be indifferent. Thus of country-bred rabbits inoculated with three milligrams human tubercle bacilli per kilogram of body weight, two of two months old showed no infection, two a year old showed caseous and miliary lesions. Again, of a blue rabbit and a Belgian hare of the same size and weight, one lived twice as long after inoculation as did the other. The author recommends, of course, much more care in future in these regards, and we shall venture to include in this charge greater humility from the bacteriologist and a recognition that science means something more than technical dexterity. It is probable that a careful inquiry into the infection susceptibility of even animals from the same litter would show individual differences to a disquieting degree. These, of course, could be got over by the use of a large material: we are naturally not condemning the search for knowledge by any and every legitimate means. But exposures such as those we have noticed above, put in conjunction with the tendency to over-rate the use of physical apparatus, as shown by the appointments to specialist posts of responsibility of those equipped merely with general pathological knowledge, make seasonable the warning just given.

* *Zur Wahl der Versuchstiere für experimentelle Tuberkulosestudien.* A. Wallgren, Upsala: Beiträge zur Klinik der Tuberkulose. May, 1921.

